# Low-dose strontium stimulates osteogenesis but high-dose doses cause apoptosis in human adipose-derived stem cells via regulation of the ERK1/2 signaling pathway

**DOI:** 10.1186/s13287-017-0726-8

**Published:** 2017-12-19

**Authors:** Abudousaimi Aimaiti, Asihaerjiang Maimaitiyiming, Xu Boyong, Kaisaier Aji, Cao Li, Lei Cui

**Affiliations:** 1 Department of Joint Surgery, First Affiliated Hospital of Xinjiang Medical University, 137 Li Yu Shan Road, Urumqi, Xinjiang 830054 People’s Republic of China; 2 Department of Urology, The First Affiliated Hospital of Xinjiang Medical University, Urumqi, Xinjiang 830054 China; 3 Department of Plastic Surgery, Institute of Medical Science, Beijing Shijitan Hospital Affiliated to Capital Medical University, 10 Tieyi Road, Beijing, 100038 People’s Republic of China

**Keywords:** Strontium, Human adipose-derived mesenchymal stem cells, Osteogenesis, ERK1/2 signaling pathway

## Abstract

**Background:**

Strontium is a widely used anti-osteoporotic agent due to its dual effects on inhibiting bone resorption and stimulating bone formation. Thus, we studied the dose response of strontium on osteo-inductive efficiency in human adipose-derived stem cells (hASCs).

**Method:**

Qualitative alkaline phosphatase (ALP) staining, quantitative ALP activity, Alizarin Red staining, real-time polymerase chain reaction and Western blot were used to investigate the in vitro effects of a range of strontium concentrations on hASC osteogenesis and associated signaling pathways.

**Results:**

In vitro work revealed that strontium (25–500 μM) promoted osteogenic differentiation of hASCs according to ALP activity, extracellular calcium deposition, and expression of osteogenic genes such as runt-related transcription factor 2, ALP, collagen-1, and osteocalcin. However, osteogenic differentiation of hASCs was significantly inhibited with higher doses of strontium (1000–3000 μM). These latter doses of strontium promoted apoptosis, and phosphorylation of ERK1/2 signaling was increased and accompanied by the downregulation of Bcl-2 and increased phosphorylation of BAX. The inhibition of ERK1/2 decreased apoptosis in hASCs.

**Conclusion:**

Lower concentrations of strontium facilitate osteogenic differentiation of hASCs up to a point; higher doses cause apoptosis of hASCs, with activation of the ERK1/2 signaling pathway contributing to this process.

## Background

Osteoporosis is a common disease with reduced bone density, and as many as half of all postmenopausal women will have an osteoporosis-related fracture during their lifetime [1]. Anti-osteoporotic drugs, including bisphosphonates and estrogen, and selective receptor-modulating drugs reduce fractures by reducing the number, activity, and lifespan of osteoclasts [2]. Compared to these anti-osteoporosis drugs, strontium ranelate (SrRan) has been shown to exert its function via promoting bone formation and simultaneously inhibiting bone resorption [3]; this was shown in several large clinical trials confirming the decreased risk of vertebral fractures in postmenopausal women with osteoporosis. SrRan may improve bone formation due to the stimulation of bone marrow stromal cell (BMSC) osteogenic differentiation. Saidak et al. [4] reported that SrRan counteracts age-related switches in BMSCs from osteoblasts to adipocytes via Wnt5a and NFATc/Maf signaling.

Mesenchymal stem cells (MSCs) are multipotent cells that have the potential for regeneration of damaged tissues in bone disease. Adipose-derived MSCs (ASCs) have several advantages, including immunoprivilege, higher yield at harvest, rapid expansion, and greater genetic stability during long-term culture [5, 6]. Evidence suggests that the administration of ASCs accelerates fracture healing and bone regeneration by direct stimulation of osteogenic differentiation or by secretion of high concentrations of cytokines.

Ca, P, Si-containing bioactive ceramic dissolution products, and SrCaPO_4_ (52.12% Sr) enhance the proliferation and osteogenic differentiation of rabbit ASCs [7, 8]. Another study [9, 10] indicated that Sr-hardystonite-gahnite (Sr-HT-gahnite), a highly porous and biocompatible Sr-zinc containing bioceramic (7.7 mg/L Sr ion), can induce osteogenesis of rat ASCs in vitro and enhance vascularized bone regeneration in vivo. However, SrRan is implicated in venous thromboembolism (VTE) and the risk of non-fatal myocardial infarction in women with postmenopausal osteoporosis [11]. Moreover, SrRan has been associated with significantly greater risk of death and cardiovascular risk compared with other osteoporotic drugs [12]. Thus, we should study the safety of SrRan with respect to osteogenic differentiation of ASCs.

Mitogen-activated protein kinases (MAPKs) regulate various physiological processes, such as cell growth, differentiation, and apoptosis [13]. Extracellular signal-related kinase (ERK)1/2 is a member of the MAPK family and is involved in the regulation of the cellular response to apoptotic-promoting signals [14]. Studies suggest that aberrant ERK activation can promote apoptosis under certain conditions, and our work suggests that osteogenic differentiation of human ASCs (hASCs) is mediated by ERK activation [15]. However, how ERK signaling functions during osteogenic differentiation of ASCs stimulated by SrRan is unclear. Thus, we studied the effect of SrRan over a range of doses on osteogenic differentiation of hASCs and assessed SrRan cytotoxicity.

## Methods

### Patients and ethics approval

Fresh human lipoaspirates were obtained from five healthy individuals (average age of 35 years) who received abdominal liposuction at the Department of Plastic and Reconstructive Surgery in Xinjiang Medical University First Affiliated Hospital (Xinjiang, China). Before surgery, all the patients offered written informed consent, and the protocols for human tissue handling were approved by Xinjiang Medical University First Affiliated Hospital.

### Cell culture

Isolation and culture of hASCs was performed according to a previous work [16]. In brief, after washing with an equal volume of 0.1 M phosphate-buffered saline (PBS; pH 7.4), lipoaspirates were digested with 0.075% collagenase type I (Washington Biochemical Corp.) at 37 °C for 60 min. The digested samples were centrifuged at 1200 × g for 10 min to obtain a high-density stromal vascular fraction (SVF), and they were resuspended in low-glucose Dulbecco’s modified Eagle’s medium (LG-DMEM) containing 10% fetal bovine serum (FBS), 100 mg/mL streptomycin, and 100 U/mL penicillin (growth medium (GM)). They were plated at 4 × 10^4^ cells/cm^2^ in 100-mm culture dishes (Falcon). When they reached 70–80% confluence, the cells were passaged and hASCs before the third passage were used in the following study.

Osteogenic differentiation of hASCs was conducted using cultures of GM supplemented with 0.01 μM 1,25-dihydroxyvitamin D3, 50 μM ascorbate-2-phosphate, and 10 mM β-glycerophosphate. For adipogenic differentiation, the cells were cultured in adipogenic medium (AM) supplemented with 0.5 mM isobutyl-methylxanthine (IBMX), 0.1 μM dexamethasone (Dex), 10 μM insulin, and 200 μM indomethacin in growth medium. The effect of SrRan on the osteogenic differentiation of hASCs was studied using Sr (0, 25,100, 250, 500, 1,000, 1500, and 2000 μM) in osteogenic medium (OM).

### Alkaline phosphatase staining and activity assay

At day 10 after induction, alkaline phosphatase (ALP) staining was performed using a leukocyte AP staining kit (System Biosciences). Briefly, the cells were fixed with formalin for 10 min, followed by incubation with BCIP/NBT working solution for 30 min in the dark at room temperature. After rinsing twice with deionized water, the cells were stained with naphthol AS-MX phosphate for 30 min in the dark. Excess dye was removed by washing with PBS three times. Positive staining of ALP was observed under a light microscope and quantified with Image-Pro Plus 5.0 software. ALP activity was measured using p-nitrophenylphosphate as the substrate. Absorbance at 405 nm was measured and the protein in cell lysates was measured using a Bradford assay at 595 nm on a microplate spectrophotometer (Bio-Rad). ALP activity was normalized according to the total protein.

### Accumulated calcium assay

On day 21 of osteogenic differentiation, the medium was removed and the cells were fixed with 70% ice-cold ethanol for 1 h, followed by incubation in 40 mM Alizarin Red S (Sigma) at pH 4.2 for 30 min at room temperature. After rinsing with fresh PBS and drying at room temperature, the calcium deposits were quantified using a QuantiChrom Calcium Assay Kit (BioAssay Systems). A comparison with an Alizarin Red S dye standard curve was used to evaluate the calcium deposition.

### Oil Red O staining

After 10 days of adipogenic induction, hASCs were stained with Oil Red O to measure the accumulation of intracellular lipid droplets. Briefly, the cells were fixed with 10% formalin for 30 min, washed in PBS, and stained with a 0.6% (w/v) Oil Red O solution for 15 min at room temperature. After rinsing with deionized water, fat droplet staining was photographed under a phase-contrast microscope (Olympus, Tokyo, Japan).

### Live/dead viability staining

Live and dead cells were measured using an assay kit (Invitrogen) according to the manufacturer’s instructions. After incubation with SrRan for 24 and 48 h, hASCs were stained with the live/dead reagent (ethidium homodimer and calcein-AM) at 37 °C for 30 min. hASC viability was analyzed under a fluorescent microscope (Nikon, Tokyo, Japan).

### TUNEL assay

To measure apoptotic hASCs, terminal deoxynucleotidyl transferase dUTP nick end labeling (TUNEL) staining was used with an In Situ Cell Death Detection Kit (Roche, Mannheim, Germany). Cultured hASCs were fixed with 4% paraformaldehyde for 1 h at room temperature, washed three times with PBS, and then permeabilized in 0.1% Triton X-100. Fixed cells were exposed to freshly prepared TUNEL reaction mixture for 1 h in the dark. Cells were then washed with PBS and observed under a fluorescent microscope (Nikon, Tokyo, Japan).

### Transmission electron microscopy (TEM)

Cells were cultured in OM medium with/without different doses of SrRan for 48 h. Harvested cells were fixed in ice-cold 2.5% glutaraldehyde for 2 h. After post-fixing in 1% OsO_4_ for 1 h, the samples were dehydrated through an ethanol series, and embedded in epoxy resin. Ultra-thin sections (60 nm) were double stained with uranyl acetate and lead citrate. Representative areas were examined under TEM (Hitachi, Tokyo, Japan).

### Quantitative real-time polymerase chain reaction (qRT-PCR)

Total RNA was isolated using Trizol reagent (Invitrogen) and 2 μg of total RNA was reverse-transcribed into 20 μl of cDNA. qRT-PCR analysis was performed using the Step One Plus Real-Time PCR System (Applied Biosystems). Primer sequences are shown in Table 1.

**Table 1:** Sequences of primers and real-time polymerase chain reaction conditions

Gene		Primer sequences
Cbfα1	Forward	5′-GTCTTACCCCTCCTACCTGA-3′
	Reverse	5′-TGCCTGGCTCTTCTTACTGA-3′
ALP	Forward	5′-ACGTGGCTAAGAATGTCATC-3′
	Reverse	5′-CTGGTAGGCGATGTCCTTA-3′
COL I	Forward	5′-TGTTCAGCTTTGTGGACCTC-3′
	Reverse	5′-CTTGGTCTCGTCACAGATCA-3′
OCN	Forward	5′-CAAAGGTGCAGCCTTTGTGTC-3′
	Reverse	5′-TCACAGTCCGGATTGAGCTCA-3′
β-actin	Forward	5′-ATCATGTTTGAGACCTTCAA-3′
	Reverse	5′-CATCTCTTGCTCGAAGTCCA-3′

*ALP* alkaline phosphatase, *Cbfα1* core-binding factor α1, *COL I* collagen-1, *OCN* osteocalcin

### Western blot

For Western blotting, extracted proteins were resolved with sodium dodecyl sulfate polyacrylamide gel electrophoresis (SDS-PAGE) and transferred to a polyvinylidene difluoride (PVDF) membrane (Millipore). After incubation in 5% bovine serum albumin (BSA) at room temperature, the membranes were incubated with primary antibody at 4 °C overnight. Horseradish peroxidase (HRP)-conjugated immunoglobulin G (IgG) was used as the secondary antibody. Immunostained protein bands were visualized with an ECL kit (CWBIO, Beijing, China). Protein was quantified and normalized to glyceraldehyde 3-phosphate dehydrogenase (GAPDH).

### Statistical analysis

Data are presented as means ± standard deviation (SD) from three experiments, and each was an average of the triplicate experiments. SPSS 16.0 software was used for statistical analysis. A paired Student’s *t* test was used to compare groups (*p* < 0.05 was considered to be statistically significant).

## Results

### Characterization of hASCs

**Fig. 1:**
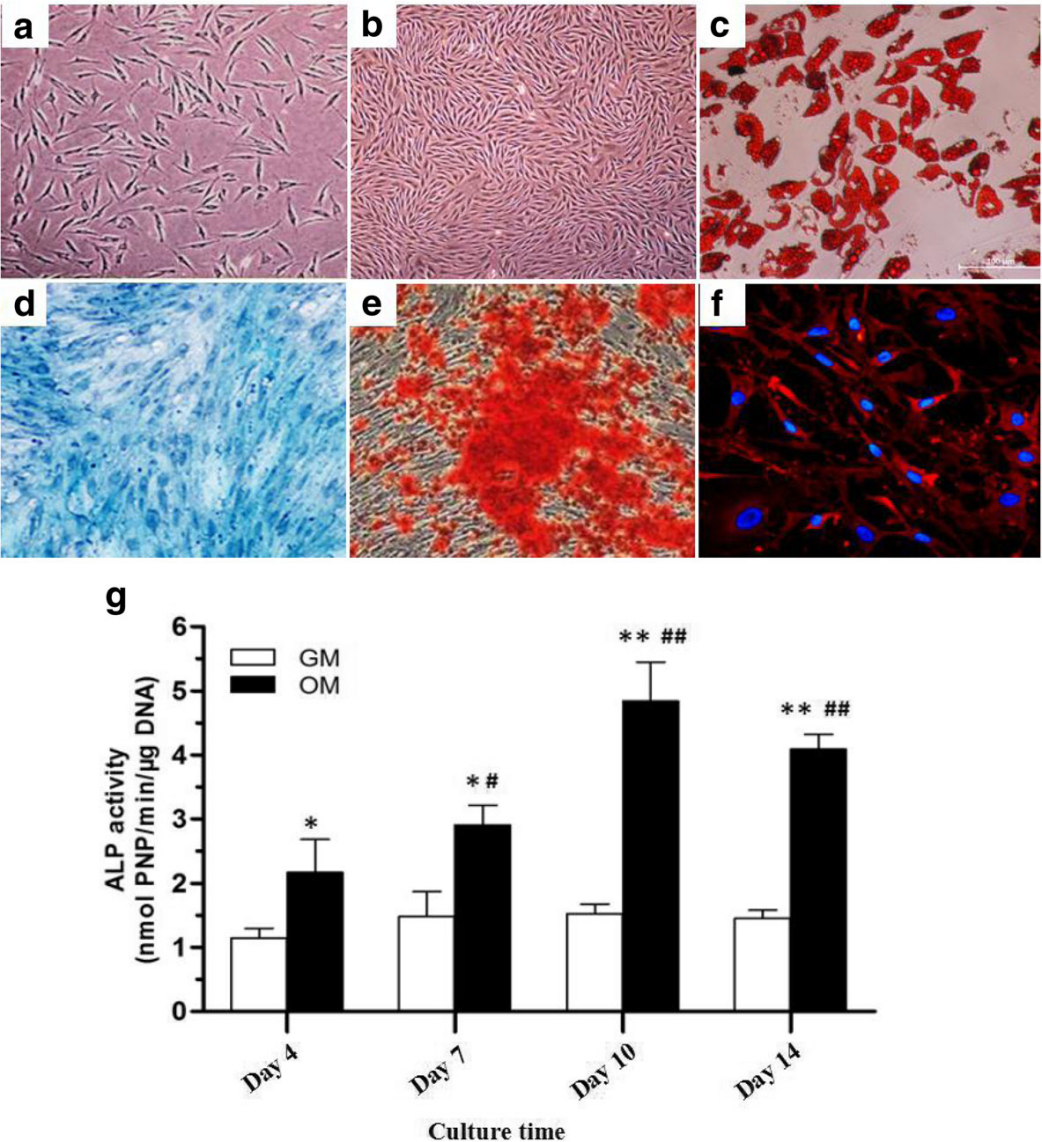
hASCs had fibroblast-like morphology as observed under a phase-contrast microscope, at passage 0 (**a**) and 3 (**b**). Adipogenic differentiation was confirmed with positive Oil Red O staining (**c**). Osteogenic differentiation was confirmed with positive ALP (**d**) and Alizarin Red staining (**e**). Chondrogenic differentiation was verified with immunofluorescent staining for collagen type II (**f**). ALP activity of hASCs at indicated days after incubation with osteogenic differentiation medium (**g**)

In the in vitro experiments, hASCs were morphologically homogeneous and fibroblast-like (Fig. 1a and b). Multiple lineage differentiation of hASCs was confirmed by osteogenic, chondrogenic, and adipogenic differentiation (Fig. 1c–f). As shown in Fig. 1g, ALP activity was significantly elevated on days 10 and 14 as indicated by ALP staining and quantitative assays.

### SrRan promoted osteogenesis of hASCs

**Fig. 2:**
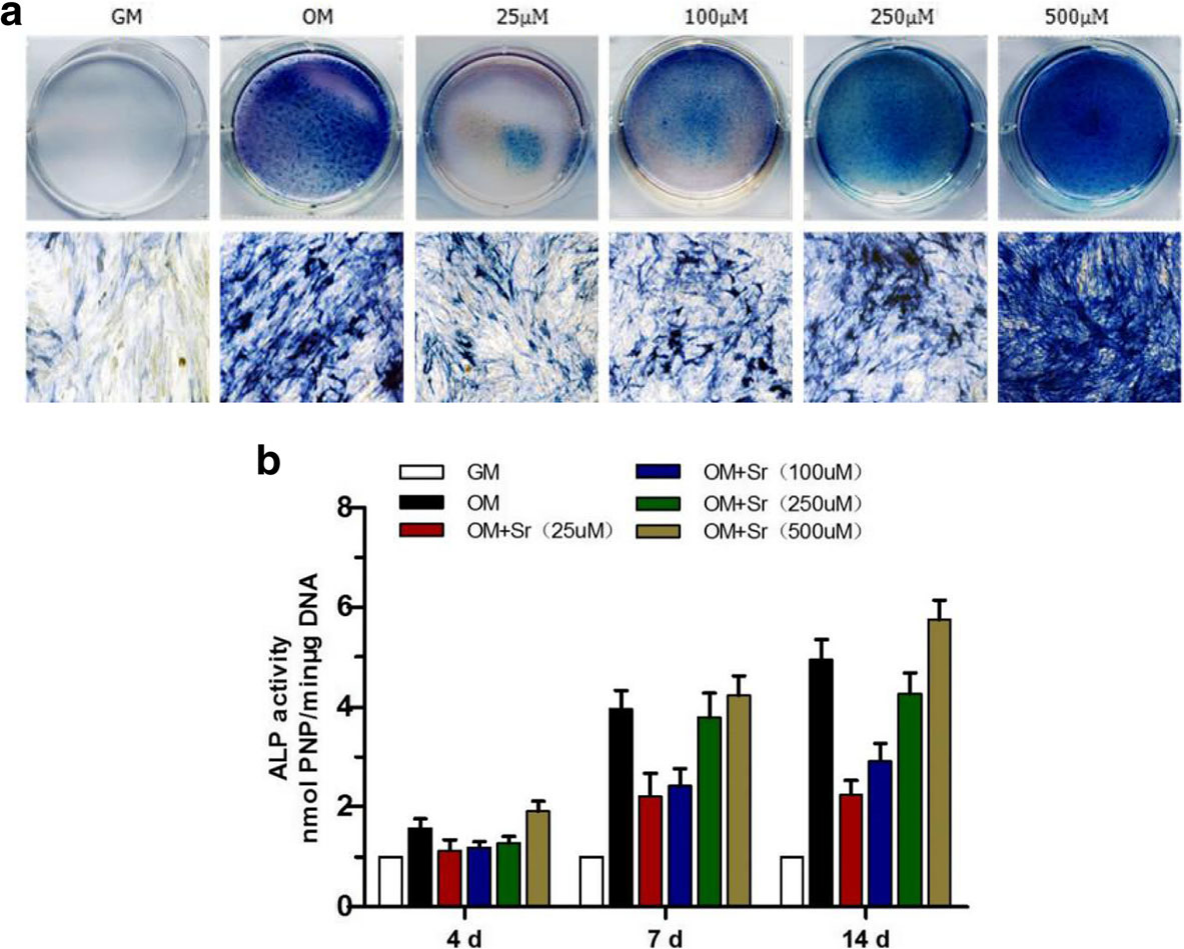
ALP staining and quantitative assay of osteo-induced hASCs in osteogenic differentiation medium with SrRan. SrRan was added at the indicated concentrations to hASCs in osteogenic differentiation medium induced for 10 days. **a** ALP expression was measured by staining. **b** Quantitative ALP activity determined with a colorimetric endpoint assay on the indicated days (*d*). *ALP* alkaline phosphatase, *GM* growth medium, *OM* osteogenic medium, *Sr* strontium

**Fig. 3:**
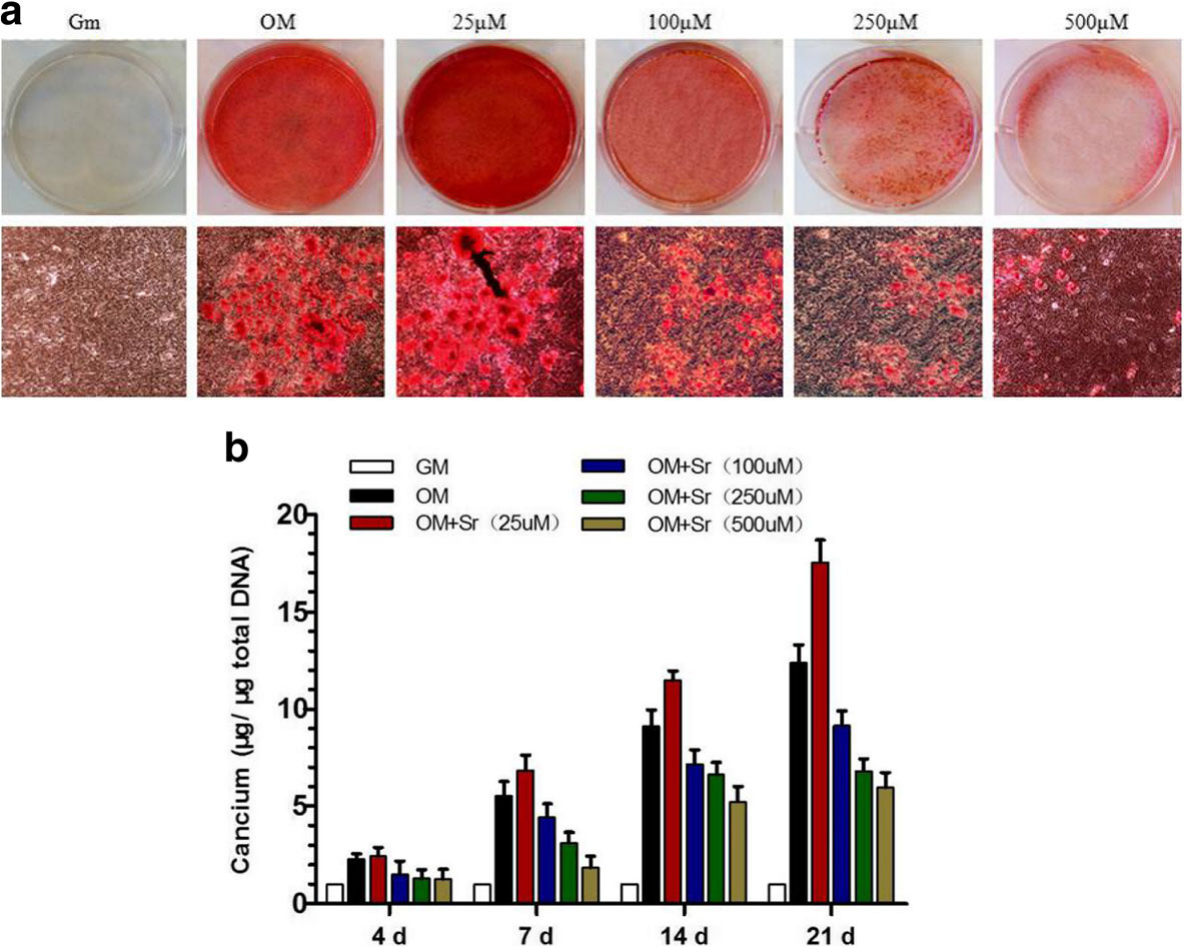
Effect of SrRan on mineralization of hASCs. **a** Effect of SrRan added at the indicated concentrations to hASCs in osteogenic differentiation medium. Extracellular calcium deposition was measured with Alizarin Red S staining at day 21. **b** Extracellular calcium deposition was quantified with a colorimetric method on the indicated days (*d*). *GM* growth medium, *OM* osteogenic medium, *Sr* strontium

**Fig. 4:**
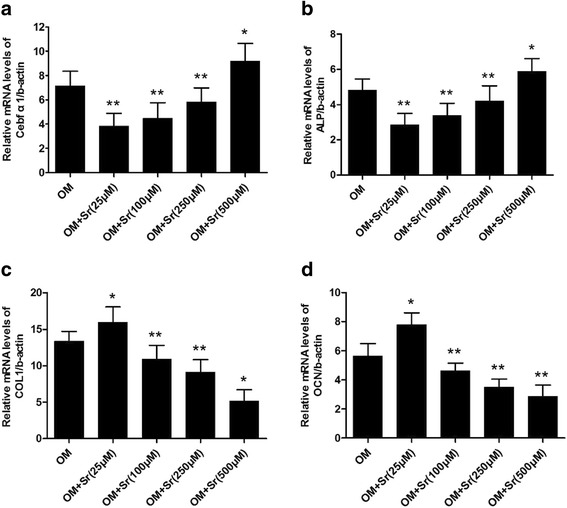
Effect of SrRan on Cbfα1/RUNX2, ALP, COL I, and OCN gene expression in hASCs. Real-time PCR for osteogenic differentiation-related gene expression of hASCs treated with SrRan (25, 100, 250, and 500 μM) in OM for 10 days. Gene expression was measured using real-time PCR. **a** Core binding factor alpha 1 (*Cbfα1*); **b** alkaline phosphatase (*ALP*); **c** collagen type I (*COLI*); and **d** osteocalcin (*OCN*). Cells cultured in OM were used as controls. *p < 0.05, **p < 0.01 vs. OM. *OM* osteogenic medium, *Sr* strontium

Figure 2a shows that SrRan (25, 100, and 250 μM) in osteogenic medium decreased ALP staining at 4, 7, and 14 days. At 500 μM, ALP staining was stronger than in normal osteogenic medium at 14 days. This result is consistent with the quantitative ALP activity data (Fig. 2b), which shows that ALP activity was suppressed at 25, 100, and 250 μM and was enhanced at 500 μM after 4, 7, and 14 days. Alizarin Red S staining showed that, at 25 μM calcium deposition was significantly increased at 14 and 21 days. At 100, 250, and 500 μM, calcium deposition was reduced (Fig. 3a and b). qRT-PCR showed that the expression of ALP and runt-related transcription factor 2 (RUNX2) was significantly reduced at 25, 100, and 250 μM SrRan, and elevated at 500 μM compared with OM at 10 and 14 days (Fig. 4a and b). In contrast, the expression of collagen-1 (COL-I) and osteocalcin (OCN) was slightly upregulated at 25 μM and was significantly suppressed at 100, 250, and 500 μM.

### High-dose SrRan induced apoptosis of hASCs

**Fig. 5:**
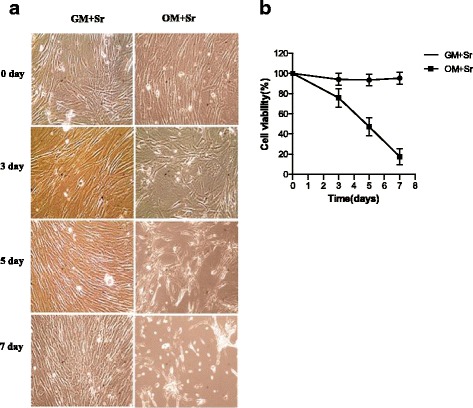
**a** Morphological features of hASCs exposed to SrRan at different time points. **b** hASCs were treated with SrRan (1000 μM) in GM and OM at the indicated days and viability was measured with a CCK-8 assay. *GM* growth medium, *OM* osteogenic medium, *Sr* strontium

**Fig. 6:**
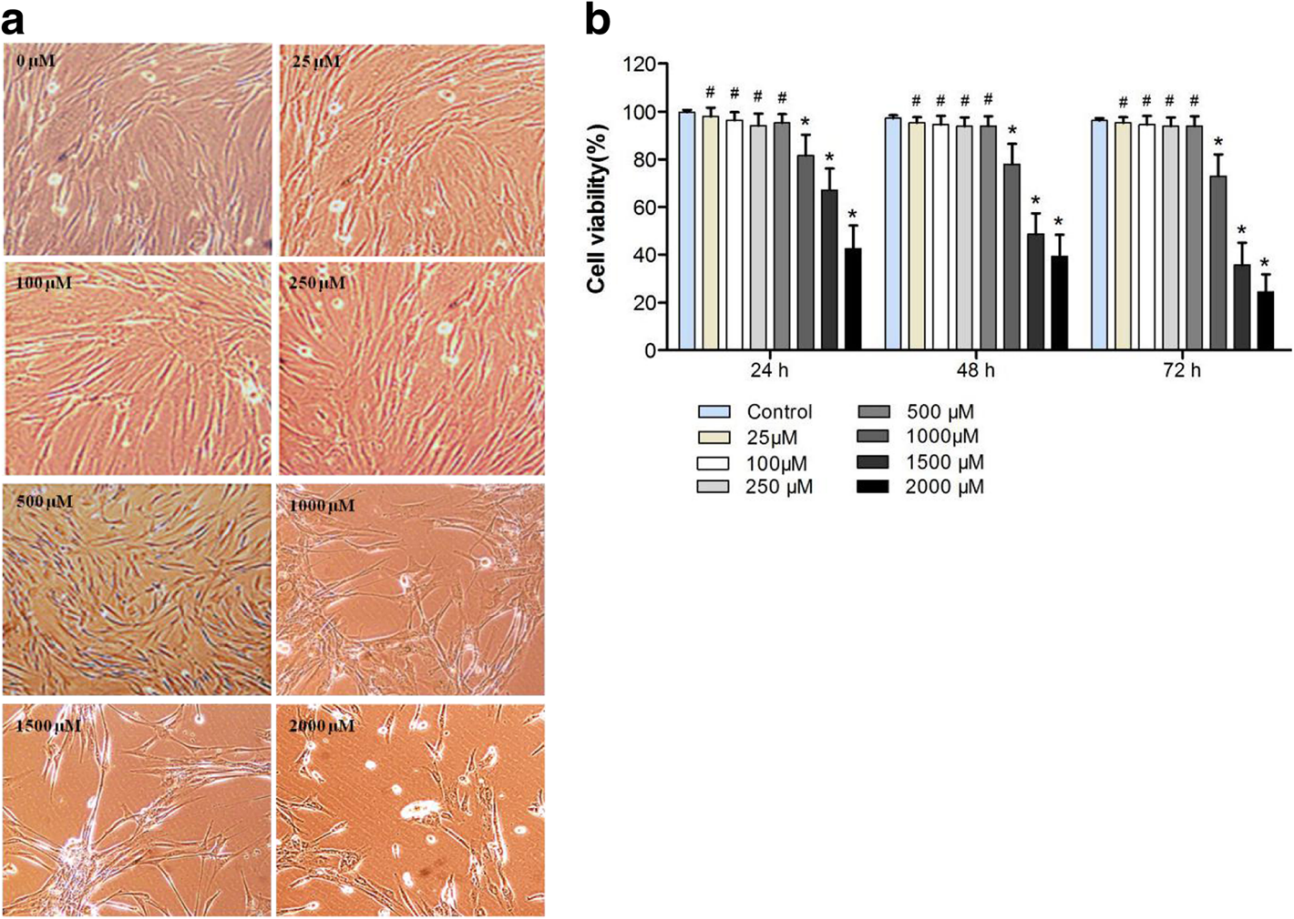
**a** Morphological features of hASCs exposed to SrRan. hASCs were treated with SrRan in GM and OM for 3 days. **b** hASCs exposed to SrRan in GM and OM, with cell viability measured with a CCK-8 assay. # *p* < 0.01 and * *p* < 0.05 compared with the control group

**Fig. 7:**
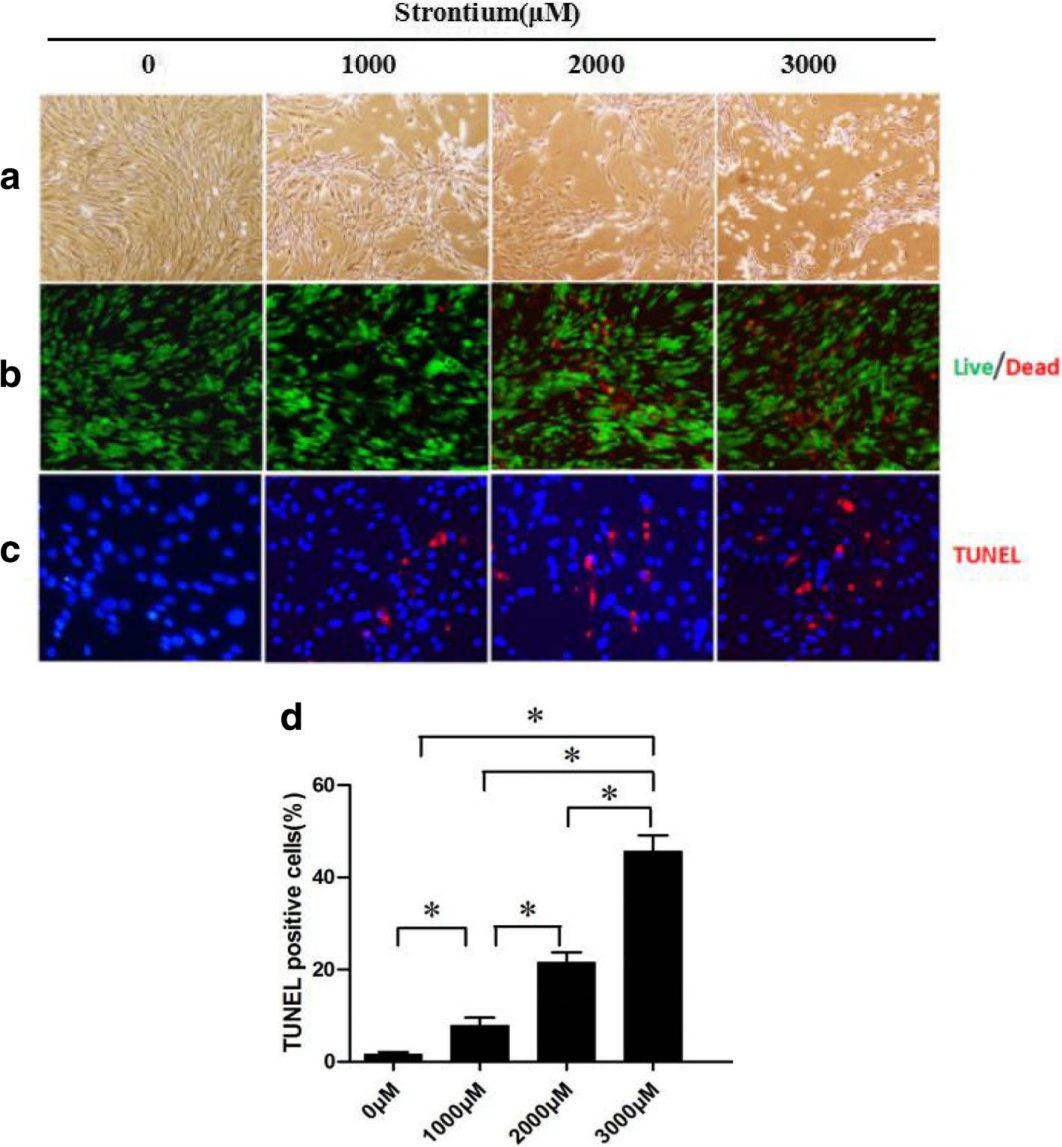
SrRan-induced apoptotic and morphological changes and decreased hASC viability. **a** Apoptotic appearance (cell shrinkage) observed in hASCs after exposure to SrRan (1000, 2000, and 3000 μM) for 48 h. **b** High-dose SrRan-treated hASCs were fewer in number compared with the controls according to live/dead staining. **c** SrRan (1000, 2000, and 3000 μM) and apoptosis of hASCs were measured with terminal deoxynucleotidyl transferase dUTP nick end labeling (*TUNEL*) staining. **d** Quantification of TUNEL-positive hASCs. * *p* < 0.05 compared with the control group

We next treated hASCs with 1000 μM SrRan for 7 days; cells growing in GM maintained their regular shape and proliferation but SrRan exposure reduced the cell number over 7 days (Fig. 5). Moreover, SrRan at 1000, 1500, and 2000 μM inhibited cell proliferation. The cell number was stable with SrRan at 25, 100, 250, and 500 μM in OM (Fig. 6). hASCs exposed to SrRan at 1000, 2000, and 3000 μM (Fig. 7a) caused shrinkage and more cells to be stained red due to apoptosis at 48 h (Fig. 7b). TUNEL assay (Fig. 7c and d) showed that SrRan at 1000, 2000, and 3000 μM for 48 h significantly increased apoptosis.

**Fig. 8:**
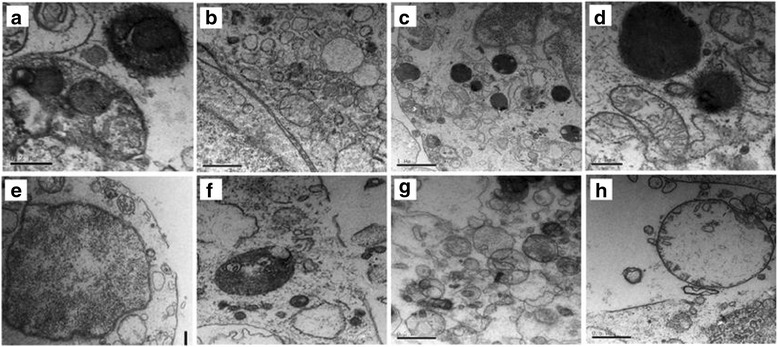
TEM analysis of hAMSC apoptosis induced by high-dose SrRan (1 mM) for 48 h. **a–d** Normal control hAMSCs. TEM of the hAMSC group showed intact cell membrane, nuclear membranes, and visible two unit-membranes (**a** and **b**); cytoplasmic organelle structures were integrated and there was a complete mitochondrial crest (**c** and **d**). **e**–**h** Apoptotic hAMSCs from the experimental groups. The cell membrane and nuclear membrane collapsed (**e** and **f**), and there was cellular content leakage, cytoplasmic organelle elimination, loss of chromatin and mitochondrial swelling, vacuolar degeneration, reduced matrix, and disappearing cristae seen with TEM (**g** and **h**). *Scale bar*s = 2 meters

TEM analysis confirmed apoptotic ultramicrostructural changes, such as cell and nuclear membrane collapse, chromatin loss, and mitochondrial swelling after SrRan (1000 μM) for 48 h (Fig. 8).

### The ERK1/2 signaling pathway is involved in high-dose SrRan-induced apoptosis of hASCs

**Fig. 9:**
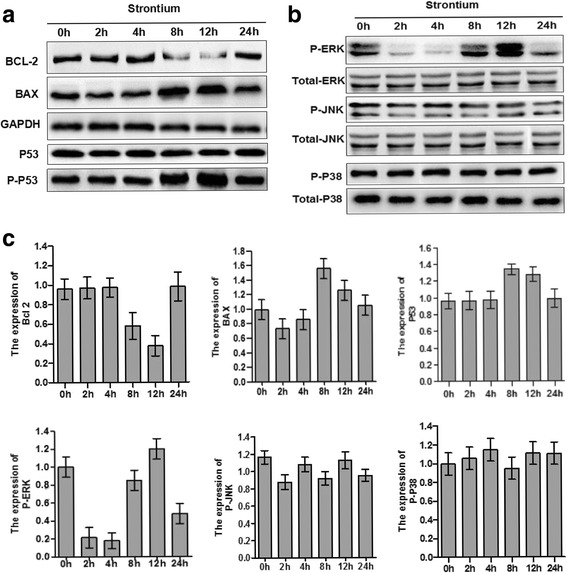
SrRan activated MAPK pathways mediated by ERK, JNK, p38, and upregulated the expression of Bax and Bcl-2 in hASCs. **a** SrRan 1000 μM treatment downregulated Bcl-2 protein, and increased the expression of BAX and phosphorylation of P53in hASCs at 8 and 12 h. **b** The expression of total and phosphorylated ERK, p38, and JNK was measured by Western blotting in hASCs treated with SrRan 1000 μM at the indicated times. Phosphorylation of ERK was significantly increased at 8 and 12 h. No change occurred with phosphorylation of p38 and JNK after 0, 2, 4, 8, 12, and 24 h. **c** Results of densitometric scans of blots. Data are means ± SEM for three independent experiments

**Fig. 10:**
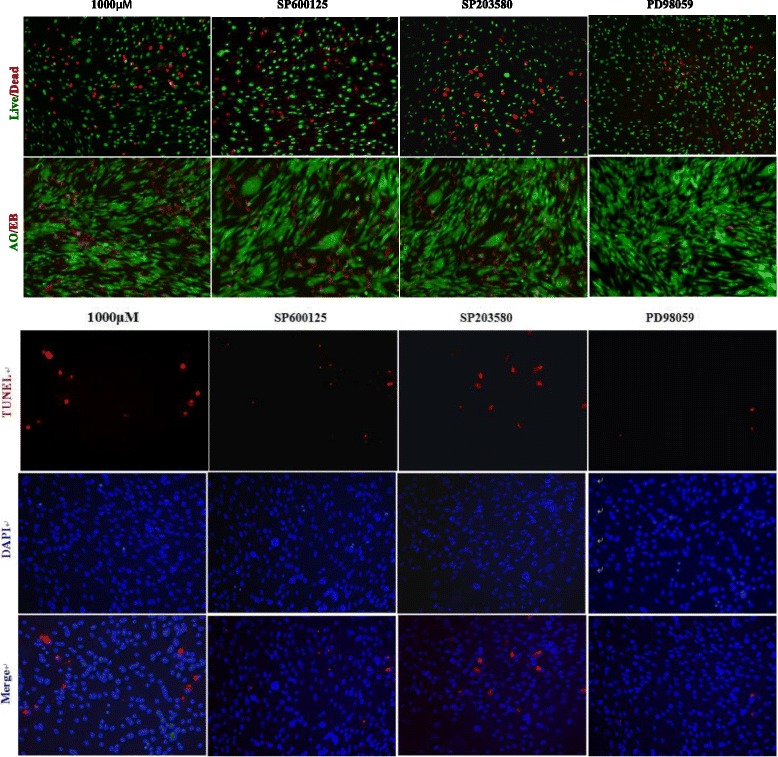
ERK signaling contributed to SrRan-induced hASC apoptosis. The apoptosis of hASCs was measured by live/dead staining, AO/EB staining, and TUNEL assays in hASCs treated with strontium 1000 μM at 48 h. The hASC apoptosis caused by high-dose SrRan treatment was greatly attenuated by ERK-specific inhibitors (PD98059); however, JNK (SP600125) and p38 (SB203580) inhibitors showed little effects on hASC apoptosis induced by high-dose SrRan

**Fig. 11:**
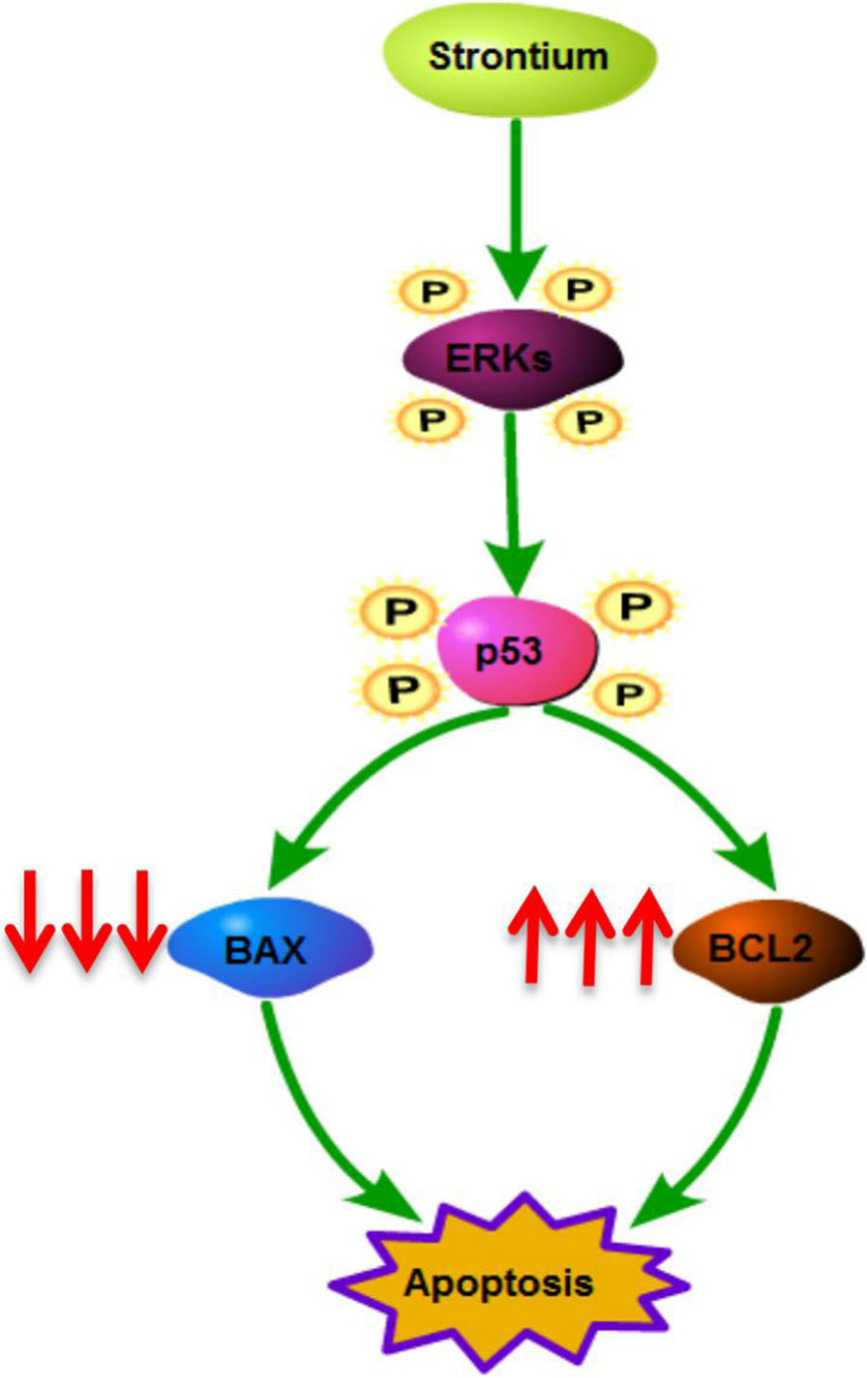
Diagram of high-dose SrRan-induced hASC apoptosis by ERK/p53-mediated pathways and subsequent Bcl-2 family-mediated mitochondrial apoptosis. SrRan activates ERK phosphorylation, which may increase phosphorylation of its target gene P53. Activation of P53, in turn, downregulates predicted target genes, anti-apoptotic protein, and Bcl-2, and upregulates the expression of the pro-apoptotic protein BAX in hASCs in OM, and causes mitochondrial apoptosis

Western blotting revealed that treatment with SrRan at 1000 μM in OM downregulated Bcl-2 and increased phosphorylation of BAX in hASCs at 8 and 12 h after induction. Moreover, Fig. 9 shows that 1000 μM SrRan treatment induced phosphorylation of ERK as early as 8 and 12 h. The phosphorylation of p38 and JNK was not observed. Consistent with TUNEL and live/dead viability staining data, the apoptosis of hASCs treated with high-dose SrRan was attenuated by PD98059 (25 μM), an inhibitor of ERK pathway activation. The specific JNK inhibitor SP600125 (10 μM) and the p38 MAPK inhibitor SB203580 (5 μM) had little effect on hASC apoptosis induced by high-dose SrRan (Figs. 10 and 11).

## Discussion

Data show that strontium promotes osteogenesis of mature osteocytes and undifferentiated MSCs in a dose-dependent manner at concentrations from 10 to 100,000 μM [17, 18]. However, the effects of low- and high-dose SrRan on osteogenic differentiation of hASCs has not been reported previously. Thus, we investigated the influence of low-dose SrRan on osteogenesis of hASCs at the concentrations used in previous reports [9]. We found that early osteogenic marker expression and ALP activity was increased at 500 μM SrRan, but it augmented late osteogenic gene expression and increased calcium deposition at 25 μM. This result is consistent with Guo et al. [19] who reported that 250–1000 μM SrRan caused osteo-induction with the greatest effect being achieved with 250–500 μM SrRan. A previous study indicated that significant increases in osteogenic gene expression occurred in MSCs cultured in OM containing 100 μM SrRan [20]. Meanwhile, previous studies showed that enhanced osteogenesis occurred in PA20-h5 cell lines treated with SrRan at 50 and 400 μM [9]. Canalis et al. [21] reported that 10 μM SrRan increased bone formation in rat calvarial cells, and another study showed a significant increase in the osteogenic marker RUNX2 in human MSCs after treatment with SrRan from 2.4–240 μM [22]. Studies have also shown that osteo-induction was achieved at 300 μM SrRan in pre-osteoblastic U-33 cells [20]. This is probably because SrRan has a biphasic effect on bone regeneration; dose-dependent effects of SrRan at different time points of hASC osteogenesis suggests a complexity of interactions of the effect of cations on bone regeneration. This is in agreement with data from Nardone et al. [9] who showed that ALP production occurred at 400 μM SrRan and calcium deposit formation occurred at lower concentrations (2.5–50 μM). Another study suggested that SrRan may inhibit calcium deposition in human pre-adipocytes, indicating that alterations of physicochemical properties in the structure of hydroxyapatite crystals impedes its formation [23]. Accordingly, our results demonstrated that SrRan (25–500 μM) promotes hASC osteogenesis in a dose- and time-dependent manner.

However, studies have also shown the opposite results, indicating that SrRan optimally simulates osteogenesis at 1000 μM or greater [24, 25]. The expression of RUNX-2 and ALP increased with SrRan greater than 500 μM, and therefore we studied the influence of SrRan at high concentrations on the osteogenesis of hASCs and whether high-dose SrRan elicited any adverse effects. We found that the growth of hASCs was significantly suppressed by SrRan at 1000, 1500, and 2000 μM, and that number of dead hASCs significantly increased at these doses. The TUNEL assay revealed that hASCs exposed to SrRan at these three doses for 48 h had more apoptotic cells. Furthermore, TEM analysis showed typical apoptotic ultramicrostructural changes in hASCs treated with 1000 μM SrRan for 48 h. To our knowledge, this is the first study to show that high-dose SrRan (>1 mM) causes apoptosis of hASCs under osteogenic differentiation. Of note, some studies show that SrRan at high concentrations promotes osteogenic differentiation of MSCs. Fromigué et al. [26] reported that 3000 μM SrCl_2_ had the greatest osteogenic induction of MC3T3-E1 cells when combined with SrRan. Peng et al. [27] demonstrated that 4000 μM SrRan increased the expression of RUNX2 and OCN in human MSCs. These differences may be explained by different cell lineages which respond differently to SrRan at similar concentrations [9, 24, 25]. Zhu et al. [20] showed that 300 μM SrRan enhanced osteogenesis of rat MSCs and pre-osteoblastic U-33 cells but it had no obvious effect on OB-6 cells. Furthermore, target genes associated with osteogenesis are variably activated by SrRan in the same cells. SrRan at 0.1–1 mM significantly upregulated BSP and OCN expression, but RUNX2 expression was not changed in osteoblast-like OB-6 cells. However, with the stimulation of SrRan at similar concentrations, the expression of RUNX2 was increased in bone marrow MSCs and OCN expression was not affected.

ERK1/2, a member of the MAPK family, is a confirmed pro-survival factor that participates in the regulation of various cell responses, including proliferation, migration, differentiation, and death [28]. ERK activation is noted in apoptosis caused by diverse stimuli [29, 30]. In our previous work, we reported ERK signaling pathway-mediated osteogenic differentiation of hASCs and adipogenic transition initiated by dexamethasone [16]. Here, we report that with the increased apoptosis of hASCs due to higher doses (≥1 mM) of SrRan, ERK was activated and the inhibition of ERK activation using PD98059 reversed hASC apoptosis. Phosphorylation of p38 and JNK did not occur and an inhibitor of p38 and JNK had no effect on hASC apoptosis. Thus, the activation of ERK signaling is responsible for high-dose SrRan-induced apoptosis of hASCs and this was not changed by the p38 and JNK pathways. Bcl-2 is a principal family of proteins that protects cells from apoptosis, and Bax induces apoptosis [31]. Thus, the balance between the pro- and anti-apoptotic Bcl-2 family of proteins influences apoptosis [32]. We found that higher doses of SrRan downregulated Bcl-2 and that the expression of BAX was increased in hASCs. Apoptosis can be initiated by intrinsic or extrinsic pathways and p53 is central to the intrinsic apoptotic pathway [33]. Thus, the phosphorylation of p53 increased in hASCs exposed to high-dose SrRan for 8 and 12 h, suggesting that SrRan-induced apoptosis of hASCs may be modulated by p53 signaling.

## Conclusions

We suggest that low-dose SrRan enhances hASC osteogenic differentiation and higher doses causes hASC apoptosis via activation of the ERK signaling pathway and subsequent Bcl-2 family-mediated apoptosis. This information may lay the foundation for developing SrRan-containing scaffolds for use in bone tissue engineering.

## Abbreviations

ALP: Alkaline phosphatase
AM: Adipogenic medium
ASC: Adipose-derived mesenchymal stem cell
BMSC: Bone marrow stromal cell
BSA: Bovine serum albumin
COL-1: Collagen-1
Dex: Dexamethasone
ERK: Extracellular signal-regulated kinase
FBS: Fetal bovine serum
GAPDH: Glyceraldehyde 3-phosphate dehydrogenase
GM: Growth medium
hASC: Human adipose-derived mesenchymal stem cell
IBMX: Isobutyl-methylxanthine
LG-DMEM: Low-glucose Dulbecco's modified Eagle’s medium
MAPK: Mitogen-activated protein kinase
MSC: Mesenchymal stem cell
OCN: Osteocalcin
OM: Osteogenic medium
PBS: Phosphate-buffered saline
PVDF: Polyvinylidene difluoride
qRT-PCR: Quantitative real-time polymerase chain reaction
RUNX2: Runt-related transcription factor 2
SDS-PAGE: Sodium dodecyl sulfate polyacrylamide gel electrophoresis
Sr: Strontium
SrRan: Strontium ranelate
SVF: Stromal vascular fraction
TEM: Transmission electron microscopy
TUNEL: Terminal deoxynucleotidyl transferase dUTP nick end labeling
